# Study of Environmental Enteropathy and Malnutrition (SEEM) in Pakistan: protocols for biopsy based biomarker discovery and validation

**DOI:** 10.1186/s12887-019-1564-x

**Published:** 2019-07-22

**Authors:** Najeeha T. Iqbal, Sana Syed, Kamran Sadiq, Marium N. Khan, Junaid Iqbal, Jennie Z. Ma, Fayaz Umrani, Sheraz Ahmed, Elizabeth A. Maier, Lee A. Denson, Yael Haberman, Monica M. McNeal, Kenneth D. R. Setchell, Xueheng Zhao, Shahida Qureshi, Lanlan Shen, Christopher A. Moskaluk, Ta-Chiang Liu, Omer Yilmaz, Donald E. Brown, Michael J. Barratt, Vanderlene L. Kung, Jeffrey I. Gordon, Sean R. Moore, S. Asad Ali

**Affiliations:** 1 Department of Paediatrics and Child Health, Aga Khan University, Karachi, Pakistan; 2 Department of Biological and Biomedical Sciences, Aga Khan University, Karachi, Pakistan; 3 Department of Pediatrics, University of Virginia, Charlottesville, VA USA; 4 Department of Public Health Sciences, University of Virginia, Charlottesville, VA USA; 5 Division of Pediatric Gastroenterology, Hepatology, and Nutrition, Cincinnati Children’s Hospital Medical Center, Cincinnati, OH USA; 6 Division of Infectious Diseases, Cincinnati Children’s Hospital Medical Center, Cincinnati, OH USA; 7 Clinical Mass Spectrometry, Division of Pathology and Laboratory Medicine, Cincinnati Children’s Hospital Medical Center, Cincinnati, OH USA; 8 Department of Pediatrics, Baylor College of Medicine, USDA/ARS Children’s Nutrition Research Center, Houston, TX USA; 9 Department of Pathology, University of Virginia, Charlottesville, VA USA; 10 Department of Pathology and Immunology, Washington University School of Medicine, St. Louis, MO USA; 11 Department of Pathology, Massachusetts General Hospital and Harvard Medical School, Boston, MA USA; 12 Koch Institute for Integrative Cancer Research at MIT and Department of Biology, Massachusetts Institute of Technology, Cambridge, MA USA; 13 Data Science Institute, University of Virginia, Charlottesville, VA USA; 14 Center for Genome Sciences and Systems Biology, Washington University School of Medicine, St. Louis, MO USA

**Keywords:** Childhood undernutrition, Low- middle income countries, Environmental enteropathy, Gut barrier function, Endoscopy, Duodenal biopsies, Small intestinal microbiota, Mucosal gene expression

## Abstract

**Background:**

Environmental Enteropathy (EE), characterized by alterations in intestinal structure, function, and immune activation, is believed to be an important contributor to childhood undernutrition and its associated morbidities, including stunting. Half of all global deaths in children < 5 years are attributable to under-nutrition, making the study of EE an area of critical priority.

**Methods:**

Community based intervention study, divided into two sub-studies, 1) Longitudinal analyses and 2) Biopsy studies for identification of EE features via omics analyses. Birth cohorts in Matiari, Pakistan established: moderately or severely malnourished (weight for height Z score (WHZ) < − 2) children, and well-nourished (WHZ > 0) children. Blood, urine, and fecal samples, for evaluation of potential biomarkers, will be collected at various time points from all participants (longitudinal analyses). Participants will receive appropriate educational and nutritional interventions; non-responders will undergo further evaluation to determine eligibility for further workup, including upper gastrointestinal endoscopy. Histopathological changes in duodenal biopsies will be compared with duodenal biopsies obtained from USA controls who have celiac disease, Crohn’s disease, or who were found to have normal histopathology. RNA-Seq will be employed to characterize mucosal gene expression across groups. Duodenal biopsies, luminal aspirates from the duodenum, and fecal samples will be analyzed to define microbial community composition (omic analyses). The relationship between histopathology, mucosal gene expression, and community configuration will be assessed using a variety of bioinformatic tools to gain better understanding of disease pathogenesis and to identify mechanism-based biomarkers. Ethical review committees at all collaborating institutions have approved this study. All results will be made available to the scientific community.

**Discussion:**

Operational and ethical constraints for safely obtaining intestinal biopsies from children in resource-poor settings have led to a paucity of human tissue-based investigations to understand and reverse EE in vulnerable populations. Furthermore, EE biomarkers have rarely been correlated with gold standard histopathological confirmation. The Study of Environmental Enteropathy and Malnutrition (SEEM) is designed to better understand the pathophysiology, predictors, biomarkers, and potential management strategies of EE to inform strategies to eradicate this debilitating pathology and accelerate progress towards the 2030 Sustainable Development Goals.

**Trial registration:**

Retrospectively registered; clinicaltrials.gov ID NCT03588013.

**Electronic supplementary material:**

The online version of this article (10.1186/s12887-019-1564-x) contains supplementary material, which is available to authorized users.

## Background

Environmental Enteropathy (EE), an acquired small intestinal condition, is a consequence of the continuous burden of immune stimulation by fecal-oral exposure to enteropathogens leading to a persistent acute phase response and chronic inflammation [1, 2]. First described in the 1960s and 1970s [3–5] in studies from Asia, Africa and Central America, morphological changes or functional signs of EE were identified in a high proportion of apparently healthy adults and children [6–9]. EE can be characterized histologically by villus shortening, crypt hyperplasia and resultant decrease in the surface area of mature absorptive intestinal epithelial cells which leads to macro- and micronutrient malabsorption [1, 10]. Concomitant intestinal leakage or permeability can be estimated by dual sugar absorption tests which have been widely used as a surrogate for biopsy based diagnoses [11, 12]. Permeability can lead to translocation of microbes or microbial products which along with the intestinal inflammatory nidus, can produce systemic immune activation. This chronic inflammation along with malabsorption are postulated to be the mechanisms through which EE contributes to undernutrition, especially linear growth faltering [13, 14]. Undernutrition is implicated in 45% of the 5 million annual deaths in children under 5 years of age [15] and linear growth failure (stunting, length-for-age Z score < − 2) is a common manifestation of undernutrition, afflicting ~ 155 million under-fives worldwide [16]. Stunting serves as a clinical marker for lifelong impairments in physical, neurocognitive, vaccine immunological response, and socioeconomic potential [17–20]. Our current understanding of EE is limited, in large part, because the tissue affected, the gastrointestinal tract of malnourished children, has been difficult to obtain in resource limited settings. Further, a comprehensive approach incorporating longitudinal surveillance of affected children to identify the impact of EE from other co-morbid conditions is needed to fully capture risk factors for EE. Therefore, we propose a comprehensive study approach which combines longitudinal surveillance of children from birth until 2 years of age, capturing known and postulated risk factors of EE and applying the most advanced tools for the analysis of the intestinal tissue samples. The Study of Environmental Enteropathy and Malnutrition in Pakistan (SEEM Pakistan), is a follow up to our phase 1 study titled ‘Identification of Novel Biomarkers for Environmental Enteropathy in Children Using an Evidence Based Approach’ [14, 21–23], in which we have studied patterns of malnutrition and prevention in a cohort of children in Matiari, Pakistan and looked at potential biomarkers of EE and at the ethical feasibilities of conducting biopsies in a low- and middle income country (LMIC) setting. Building on our experience from this study and related works [14, 21–23], our SEEM Pakistan study is designed to better understand the pathophysiology, predictors, biomarkers, and potential management strategies of EE. This report describes the SEEM Pakistan study design, including the materials that are being collected along with proposed analysis including use of machine learning methods.

## Methods

SEEM Pakistan is a multi-institutional collaboration between the Aga Khan University Hospital (AKUH), Pakistan, University of Virginia (UVa), Cincinnati Children’s Hospital Medical Center (CCHMC) and Washington University in St. Louis (WUSTL) in the USA, with funding by the Bill and Melinda Gates Foundation (2016 through 2019). Enrollment has been completed and a cohort of 400 children has been established (350 malnourished children and 50 well-nourished healthy controls).

### Objectives

This study aims to (i) establish a cohort of 350 malnourished and 50 well-nourished children in Matiari, Pakistan aged zero to 6 months; (ii) assemble serum, fecal, and urine samples for assessment as biomarkers of EE; (iii) provide educational and nutritional interventions according to the level of malnutrition of the child; (iv) evaluate the subset of malnourished children who fail to respond to educational and nutritional interventions by upper gastrointestinal (UGI) endoscopy to identify treatable causes of malnutrition; (v) use the UGI biopsy specimens obtained for detailed assessment of histopathology, gene expression and immune profiling to better characterize the pathophysiology of EE, validate current candidate biomarkers, and discover novel biomarker candidates. Importantly, this study provides a unique opportunity to examine whether there are identifiable relationships between histologically-diagnosed EE and the configuration of the proximal small intestinal and fecal microbiota. Moreover, preclinical tests of causality will be performed by transplanting bacterial communities recovered from children with EE into gnotobiotic mice and assessing the degree to which these communities transmit histopathologic, transcriptional, proteomic and immunologic features of the children’s gut barrier dysfunction phenotypes. With these goals in mind, SEEM is comprised of two primary sub-studies: 1) Longitudinal analyses of growth in birth cohort members and 2) Correlating ‘omic phenotyping with biopsy analysis, including correlating gut microbial community features with features of duodenal mucosal gene expression profile and immune phenotypes. Table 1 further describes these two primary sub-studies, including objectives covered under each study, their hypotheses, and the patient population selected for each objective.

**Table 1:**
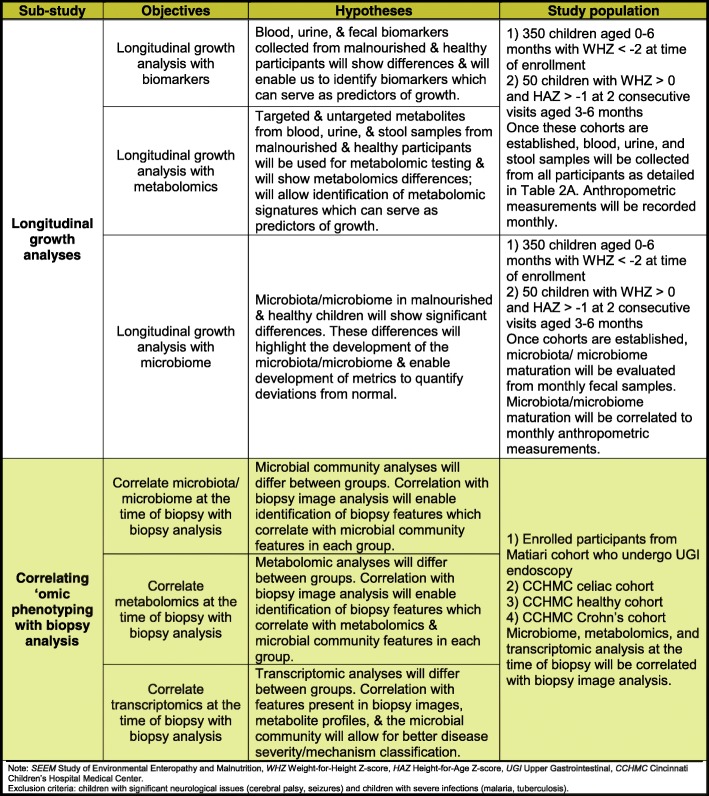
Objectives, hypotheses, and study population of the primary SEEM sub-studies

### Patient and public involvement

Our enrollment population consists of children under the age of 2 years. Therefore, not the patients themselves but their parents were indirectly involved in certain aspects of SEEM’s study design. Our field study staff has ongoing routine feedback and evaluation with the parents of the patients, and the current design was evolved based upon our and patient/parental experience from our phase 1 EE study [14]. For example, due to a parent-reported increase in their child’s diarrhea after consumption ready-to-use therapeutic food, we replaced it with a locally made supplement (Acha Mum) in the current study. Furthermore, feedback from the parents is encouraged, and all issues and comments are communicated to the study team during weekly community meetings. We plan to disseminate results to each participant/parent at the Matiari field-site office at the end of the study.

### Study settings and participants

**Fig. 1:**
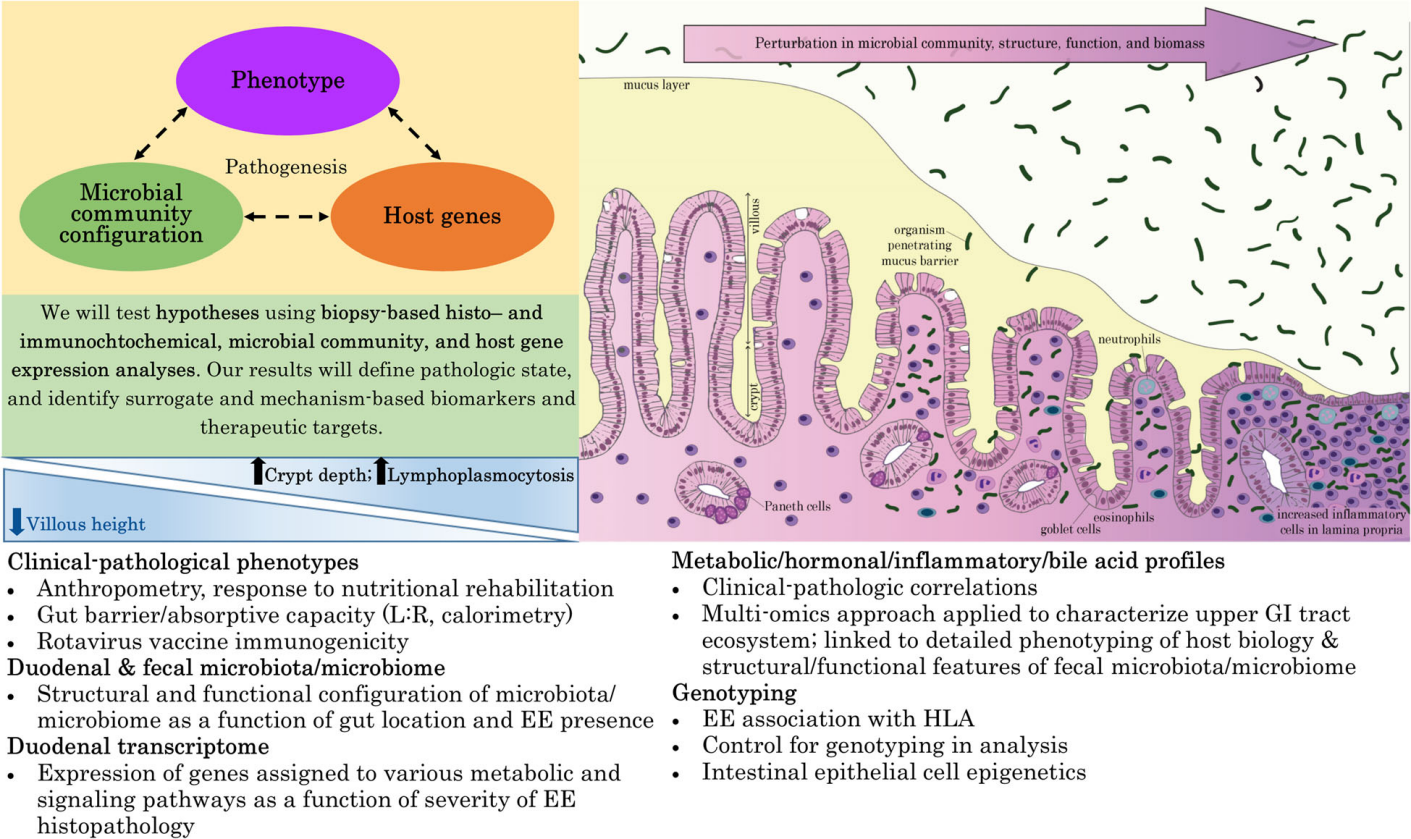
Conceptual framework for hypothesis testing in SEEM. The severity of clinical phenotypes in Matiari children with wasting and suboptimal response to nutritional rehabilitation will highly correlate with the histopathological appearance of duodenal biopsies; duodenal and fecal dysbiosis; perturbation of duodenal gene expression profiles; systemic biochemical profiles; and children’s genotypes. The image in the top right panel demonstrates the histological changes observed in the small intestine as environmental enteropathy progresses. Note: *L:R* lactulose:rhamnose ratio, *EE* environmental enteropathy, *GI* gastrointestinal, *HLA* Human Leukocyte Antigen

**Fig. 2:**
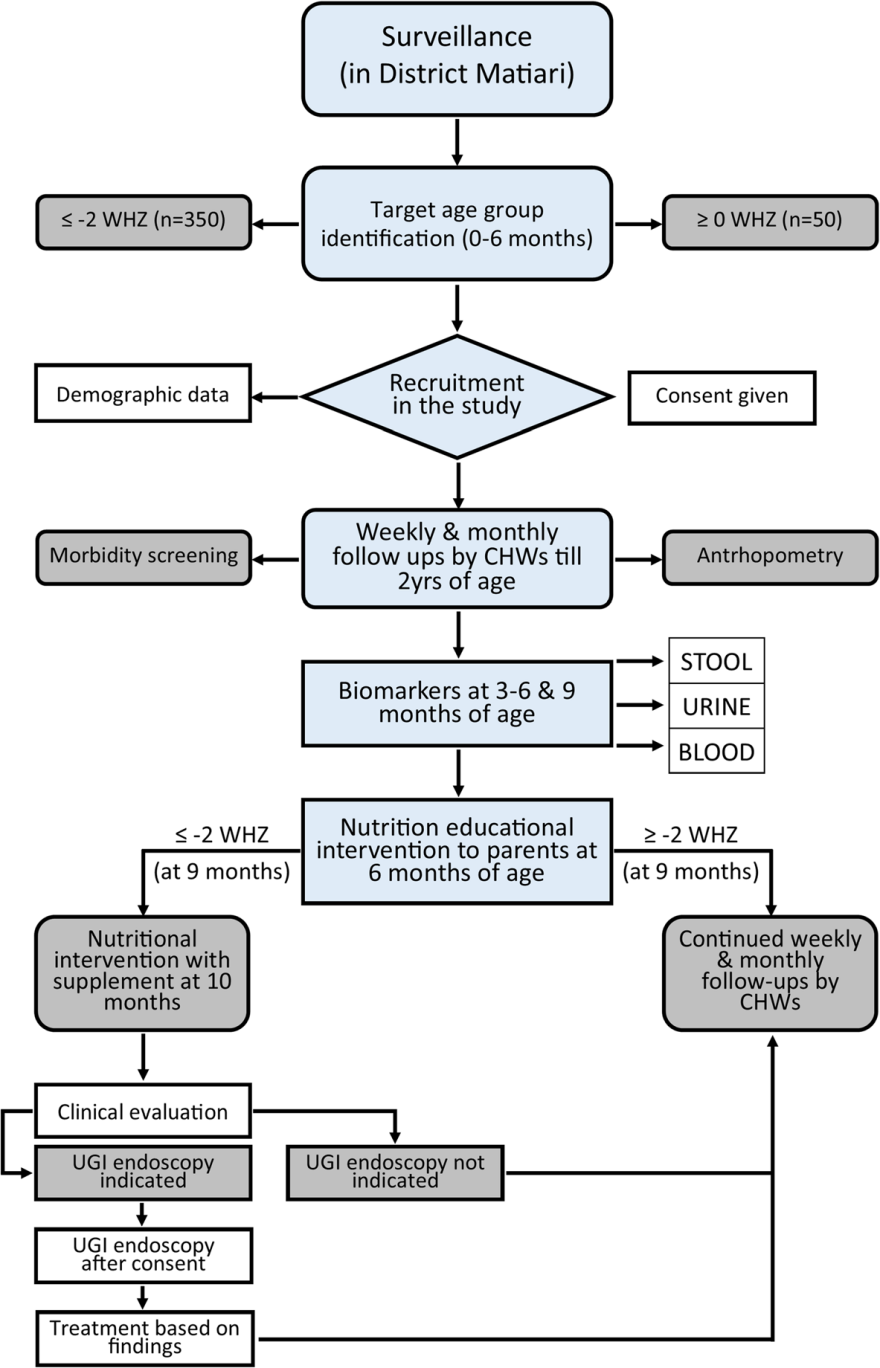
SEEM Data Collection Process. Note: *CHW* community health workers, *UGI* upper GI, *WHZ* weight for height Z score

The basic framework for the SEEM Pakistan study is described in Figs. 1 and 2. The Department of Paediatrics and Child Health at AKUH has an established field site at Matiari, Pakistan, which is a rural district about 3 hours drive north of Karachi, Pakistan. We anticipated enrolling 350 children from ages 0 to 6 months with weight for height Z score (WHZ) < − 2 at the time of enrollment. We also anticipated enrolling 50 children of the same age with healthy ponderal and linear anthropometric assessments based on consistent WHZ > 0 and height for age Z score (HAZ) > − 1 on two consecutive visits between 3 to 6 months, to serve as healthy controls. Administration of routine rotavirus vaccine will be facilitated as a part of our study, and other Expanded Program on Immunizations (EPI) vaccines will also be facilitated as part of other ongoing research activities.

Blood, urine, and fecal samples will be collected from all participants between 3 and 6, and at 9 months of age as well as at the time of endoscopy for those who undergo the procedure (Table 2). Lastly, feces will be collected from participants eligible for nutritional intervention at 10 months of age (pre-intervention) and then again at around 14 months of age (post-intervention). Duodenal aspirates will also be collected at the time of endoscopy; a dry aspirate (pre-saline lavage) as well as a wet aspirate (post-saline lavage) will be attempted.

**Table 2:**
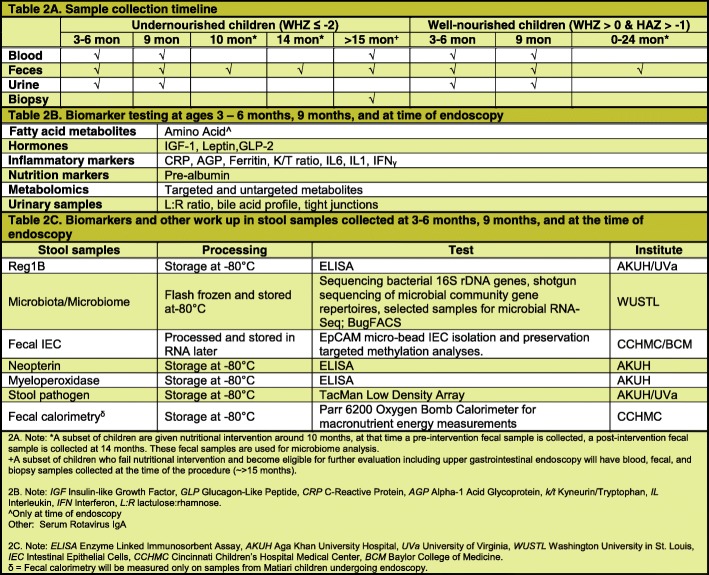
Description of sample collection

After enrollment, the parents/caregivers of all participants will undergo a series of rehabilitative interventions to improve the child’s nutritional status. Those participants who remain moderately or severely malnourished (WHZ < − 2 or < − 3, respectively) despite interventions will then be eligible for medical evaluation to assess if s/he merits further clinical workup of malnutrition, including UGI endoscopy, to identify a secondary cause. Those who qualify for UGI endoscopy will also undergo a biopsy workup as described in Table 3.

**Table 3:**
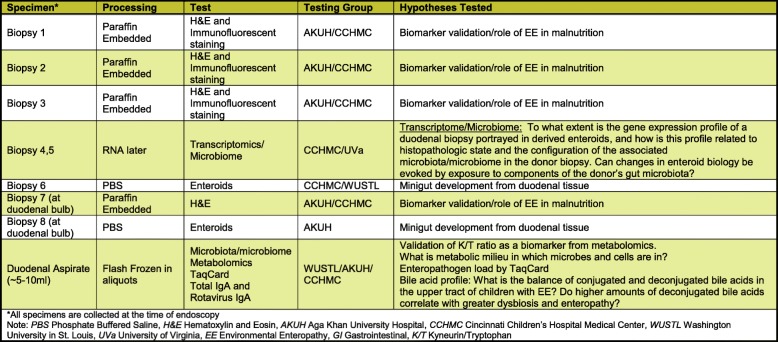
Plan of biopsy work up on children selected for UGI endoscopy

Because UGI endoscopies are rarely performed in children under 2 in Pakistan and due to ethical and cultural considerations, it is not possible to obtain duodenal biopsies from national healthy children that could serve as a control in our analysis. This is one of the major limitations of our study and as the results from Campbell et al. [10] support the utility of healthy age-matched children from high-income countries for identifying key gut pathogenic pathways in low-income settings, we are proposing to use age-matched controls from the United States for comparison.

Therefore, we plan to enroll 3 different control groups, all comprising of children under the age of 11 years, with a preference to enroll children under the age of 5 years. Our first control group will comprise of 30 healthy children, who will undergo endoscopy at CCHMC as part of a diagnostic workup for digestive symptoms, but whose biopsies and diagnoses are not supportive of eosinophilic esophagitis, celiac disease, or inflammatory bowel disease, and who were not treated with antibiotics ≤4 weeks prior to endoscopy.

As EE and celiac disease share some shared histopathological features [24, 25], we will focus on celiac disease as an enteropathy control group. We plan to enroll 30 children with newly diagnosed celiac disease per endoscopy at CCHMC to assess the extent to which gene signatures and associated biologic pathways for children with celiac disease or EE overlap or differ. Lastly, while we recognize that duodenal and ileal histopathology in the majority of Crohn’s cases differ from celiac disease and EE, the differentially expressed ileal gene signature in Crohn’s patients bears remarkable similarities to individual gene expression patterns reported for EE [10, 26], i.e. induction of IFN_γ_, REG1B. Therefore, our third control group will be 30 children with newly diagnosed Crohn’s disease per endoscopy at CCHMC.

### Sample size

Participants will be recruited from our prospective cohort. Based on our phase 1 cohort [14], we anticipated that the cohort of 50 SEEM patients, with duodenal samples collected by endoscopy, will include at least 20 without a single identified treatable infection, i.e. identified *Giardia* or *H. pylori* infection*.* The primary endpoint guiding our sample size estimate will be the anticipated differences in duodenal IFN_γ_ and APOA1 gene expression between subjects with EE and healthy controls. We anticipate that induction of IFN_γ_ gene expression will be associated with a reduction in APOA1 gene expression as per a recent study on Crohn’s disease [27] and that of Bragde et al. on celiac disease [28]. In the Crohn’s study, the mean (SD) Reads Per Kilobase per Million Mapped reads (RPKM) IFN_γ_ gene expression at diagnosis was equal to 1.86(2.7) in patients with Crohn’s, and 0.33(0.38) in healthy controls. The mean (SD) RPKM APOA1 gene expression at diagnosis was 927(1469) in patients with Crohn’s, and 3012(3080) in healthy controls. We anticipate similar differences between EE and healthy controls in our study. Based on these results, 30 healthy controls and 25 EE subjects without a specific treatable infection will provide 90% power to detect such a difference with α = 0.05. The secondary endpoint will be to perform an undirected analyses to capture the overall gene and signatures that are different between described groups. Based on previously published data for RNA-Seq samples size estimation [29], if we estimate a coefficient of variation of counts of 0.4 as was observed in 90% of the genes in a range of human studies, alpha of 0.05 and power of 0.8, a sample size of 20 per group will be needed.

### Educational and nutritional interventions, and steps following failure

Upon enrollment at age < 6 months, infants will be started in a 4-week home delivered educational program that will focus on breast feeding and complimentary feeding. Counseling will be performed by the study staff using standardized teaching materials. Compliance to the instruction will be recorded during weekly home visits.

If the WHZ remains < − 2 by 9 months of age despite the initial educational counseling, s/he will be enrolled in the second phase of nutritional and educational intervention. Families will be shown a 10 min educational video that details the best practices with respect to complimentary feeding best practices on a fortnightly basis, and compliance to the instructions will be recorded during the weekly home visits. If the child remains at WHZ < − 2, s/he will undergo the third phase of rehabilitation management according to Pakistan’s Community Management of Acute Malnutrition protocol [30]. This will include provision of Acha Mum for the treatment of moderate and severe acute malnutrition to the child at home with close follow up. Utilization of the food supplement will be monitored closely by weekly home visits.

For those children who fail to respond to nutritional rehabilitation and in whom no apparent cause of malnutrition can be identified after basic laboratory workup, we will conduct a more thorough investigation to identify the cause of undernutrition. It is important to note that this nutritional rehabilitation program is immensely supervised, with study staff allowed to visit homes more than once a week to ensure compliance if required. Additionally, our Phase 1 study had a 90.5% compliance for nutritional intervention, and we will therefore be able to identify which children fail to respond to rehabilitation due to biological reasons vs the unlikely event of failing due to a lack of compliance. If the child remains at WHZ < − 2 despite all the above interventions, then s/he will undergo medical evaluation (including a core standardized laboratory panel which includes celiac screening, complete blood count, complete metabolic panel, international normalized ratio, erythrocyte sedimentation rate, and C-reactive protein, additionally, the pediatric gastroenterologist will not be limited to this panel and may order any additional tests as clinically indicated) to assess if there is a clinical indication for further workup to identify a secondary cause of the malnutrition, including UGI endoscopy at AKUH. Diagnostic evaluation, including the UGI endoscopy, will be used to guide further management. For example, dietary management of celiac disease counseling (i.e. initiation of gluten free diet [31]), will be provided to the families of affected children in the local language, and identified infections will be treated according to the standard of care (as was performed in our Phase 1 study for n = 1 child diagnosed with celiac disease [14]). Follow-up will be continued to facilitate treatment and ensure the best possible outcomes depending on the pathology identified.

### Collection, preparation, storage and transport of biologic samples

Blood, urine, feces, biopsy tissues, and aspirates will be collected, prepared, preserved, and transported according to the standard operating procedures prepared for this protocol. Samples will be collected at the time points aforementioned.

Community health workers (CHWs) will be responsible for the collection of fecal and urine samples (Additional file 1: Figure S1). Urine samples will be aseptically collected into 100 mL pediatric urine collector bags using a suprapubic tap, 2 mL of urine will be aliquoted in a 4.5 mL cryovial and stored at -2 °C or -8 °C during transport to the Matiari lab and then to the Pediatric Infectious Diseases Research Laboratory (IDRL) at AKUH, once at the IDRL the urine samples will be stored at − 80 °C.

Fecal samples will be collected and cryopreserved within 30 min of production and then transported to the Matiari lab. At the lab, cryovials will be placed into a -80 °C freezer prior to shipping on dry ice to Washington University in St. Louis (WUSTL), USA. Approximately 1 g of fecal material per unique sample/time-point is required. No additives, preservatives or media will be added to the fecal samples.

For blood samples, trained phlebotomists will collect 3-5 mL of venous blood in a labeled blood collection tube (neutral vacutainer tube) after following all aseptic precautions. After collection, the labeled tube will be held upright in a test tube rack for 30 min to allow the blood to clot at room temperature. Each sample will be centrifuged for serum separation and then after successful separation will be pipetted into labeled cryovials. This initial processing will be done at our field site research lab. The vials will be stored in a cooler maintained at 2-8 °C during transportation to the Pediatric IDRL at AKUH, where they will be stored at − 80 °C freezers.

Screening for celiac disease will be performed via testing for serum TTG-IgA. We will also screen for the most important determinant of genetic susceptibility for celiac disease i.e. the presence of human leukocyte antigen-DQ (HLA-DQ) heterdimers DQ2 and DQ8 using Genome Wide Association Studies (GWAS). For participants undergoing UGI endoscopy, gastric biopsies (from the antrum and body) will only be obtained at the discretion of the pediatric gastroenterologist performing the endoscopy. These biopsies will be microscopically assessed for *Helicobacter pylori* associated gastritis on hematoxylin and eosin (H&E) stain, and a duodenal biopsy will also be microscopically assessed for the presence of *Giardia* on H&E stain. The plan for the biopsy workup is detailed in Table 3.

### Environmental enteric dysfunction biopsy initiative (EEDBI) consortium and EE score

The EEDBI Consortium [32] has been assembled from Bill and Melinda Gates Foundation EE biopsy funded projects with cohorts in Zambia [33], Bangladesh [34], and Pakistan. Recently a preliminary EE score, which incorporates acute and chronic inflammation, the presence of inflammatory cells, villus architecture, secretory cells, enterocyte injury, and epithelial detachment, is under development by the consortium, and a preliminary construct was used in our Phase 1 work [22]. The final biopsy scoring system is being developed by a team of pathologists and will be an extension of this preliminary scoring system. We will be using this score on our duodenal biopsies to assess the spectrum of EE.

### Biomarkers

In our Phase 1 work, we noted significant associations between several biomarkers and longitudinal Z scores for subsequent child height and weight  [17, 21, 26]. These biomarkers in addition to an expanded biomarker panel have been selected to test for intestinal barrier structure and function in SEEM. Data will be collected in a longitudinal fashion; monthly anthropometric measurements over 18 months, and biomarker assessment at the aforementioned time points. The biomarkers to be tested in blood and feces are listed in Table 2.

### Fecal calorimetry

In those children who undergo endoscopy at AKUH, fecal calorimetry (6200 Isoperibol Calorimeter; Parr Instrument Company, Moline, IL, USA) will be performed to obtain macronutrient specific determination of fecal energy [35]. Total protein, fat, and carbohydrate energy content of a single fecal aliquot will be compared against the child’s clinical phenotype (including severity of wasting), fecal and duodenal enteropathogen burden, endoscopic inflammation, and histologic severity.

### Fecal intestinal epithelial cells

Isolation and characterization of intestinal epithelial cells (IEC) from feces as a “liquid biopsy” for epigenetic-based detection of colorectal cancer has become an area of intense study [36, 37]. We have adapted these emerging technologies to EE, such that the isolation and preservation of exfoliated IECs from fecal specimens (fecal samples taken at 3–6 and 9 months, and additionally from children undergoing UGI endoscopy 48 h prior to the procedure) is currently in process to allow for assessment of targeted IEC DNA methylation as a function of age, growth, microbiome and enteric illnesses.

### Lactose/Rhamnose (L:R) test

The L:R test is a promising functional test that reflects gut permeability and absorptive capacity [38, 39]. This test is currently being validated in multiple field settings via the EEDBI Consortium [40] and has shown to be more advantageous compared to the lactulose/mannitol test (more often reported dual sugar permeability test in the past two decades) due to lack of pre-dose urinary rhamnose in comparison to mannitol which is used as an inactive ingredient in some oral vaccines and in foods [41]. Dual sugar permeability testing has been used as a surrogate marker of EE [42, 43]. Since our study provides an objective, histology-based diagnosis of EE, we will perform the L:R test in all children (malnourished as well as healthy controls) at approximately 13 months of age. The goal of this is to assess in children who fail to respond to nutritional intervention, whether this failure is associated with an alteration in their intestinal permeability. We will then correlate the findings of the L:R test with the histology of UGI mucosa in malnourished children.

### Gut microbiota/microbiome

Recent work that combines (i) culture-independent analyses of fecal samples collected from healthy members of birth cohorts living in Bangladesh and Malawi with (ii) machine learning algorithms have defined a normal program of gut microbial community development [44–46]. This program is manifested by temporal changes in the representation of ‘age-discriminatory’ bacterial strains. Applying this microbial signature of normal community assembly (maturation) to children diagnosed with severe acute malnutrition (SAM) revealed that their microbiota appear younger than those of their chronologically age-matched healthy counterparts living in the same locale [44, 45]. Moreover, transplantation of microbiota from healthy and undernourished children into young germ-free mice has provided preclinical evidence that gut microbiota immaturity is causally related to many of the manifestations of undernutrition [44–46].

To date, studies of the role of the gut microbiota in the pathogenesis of environmental enteropathy (EE) have been limited by challenges in obtaining well-preserved upper GI communities from individuals whose disease status has been confirmed by endoscopic evaluation. To define the relationship between the configuration of the fecal microbiota and histopathologically-defined EE in the SEEM cohort, we will first generate Random Forests-derived models of normal gut microbial community development; this will be done using bacterial V4-16S rDNA and shotgun sequencing datasets of community DNA respectively, generated from monthly fecal samples collected for the first 2 years of life from well nourished children (WHZ > 0 and HAZ > -1) of the Matiari birth cohort. These culture independent methods will be applied to duodenal biopsies and aspirates collected at endoscopy from children who failed to respond to nutritional intervention in order to identify bacterial strains (and members of other domains of life and their viruses) in the proximal small intestine whose representation/abundance are correlated with severity of EE (as assessed by histologic grading [22]). Bacterial strains will be cultured and their genomes sequenced. BugFACS [47] will also be performed on fecal samples obtained from children at the time of endoscopy to identify bacterial strains whose targeting by mucosal IgA is correlated with pathologic features of disease. These results will be further contextualized using data obtained from (i) multi-omics analysis of duodenal specimens from the same children that will be performed at CCHMC and WUSTL (RNA-Seq, metabolomics/proteomics), plus (ii) results of EE biomarker analyses performed on contemporaneously collected plasma and fecal specimens.

A follow-on component of this work will involve transplantation of duodenal microbial community members collected from children with varying degrees of EE severity into germ-free mice fed a prototypic diet consumed by children living in Matiari. The objective will be to test the hypothesis that these communities transmit enteropathy to recipient animals and the relationship between enteropathy features and growth faltering/undernutrition. These assessments include measurements of (i) lean body mass gain (quantified by whole body magnetic resonance), (ii) bone growth (measured by micro-computed tomography and by serum biomarkers of osteoblastic and osteoclastic activity), (iii) gut barrier function (histochemical and immunohistochemical markers such as EpCAM, claudin-2/− 4, tight-junction protein-1, functional assays such as Fluorescein Isothiocyanate (FITC)-labelled dextran permeability, transcriptional (RNA-Seq)/proteomic analyses of different gut segments, and (iv) immune phenotypes (FACS sorting of intestinal and extra-intestinal tissues). If preclinical proof of concept is established for a causal role of the small intestinal microbiota in the pathogenesis of EE, these gnotobiotic models will permit a search for key effector microbes, the mechanisms through which they operate and ultimately tests of therapeutic concepts.

### Histopathologic, immunohistochemistry and transcriptomic work up of biopsy specimens

We hypothesize biopsies from children whose endoscopic workup does not reveal a clear malabsorptive pathology, such as celiac disease, will likely demonstrate advanced features of EE. Previous studies suggest at least two factors contribute to EE: (i) chronic T-cell mediated intestinal damage and (ii) perturbations in microbial community structure/function [13, 48]. Recognizing that our sample will be restricted to children whose wasting (WHZ ≤ − 2) is refractory to nutritional intervention, we will comprehensively assess biopsy specimens to better understand the pathology of the proximal small intestinal mucosa in EE.

**Fig. 3:**
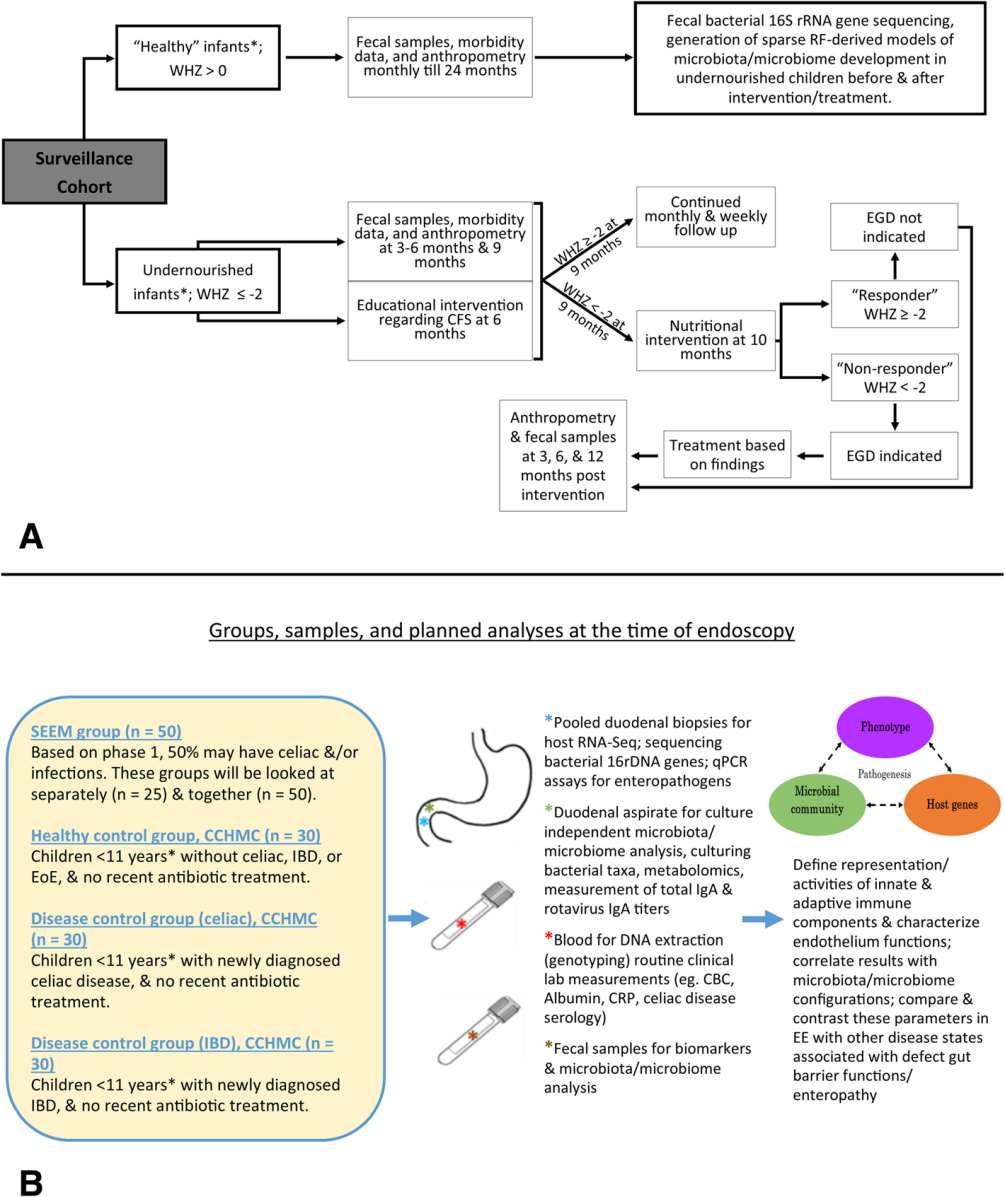
SEEM transcriptome/genetics/biomarkers/microbiome framework at the time of endoscopy. **a** Is a detailed description of how samples will be collected throughout the study process from the birth cohorts; **b** Shows the groups, samples, and planned analyses at the time of endoscopy. Note for **a**: *WHZ* Weight-for-Height Z score, *RF * Random Forest, *EGD* Esophagogastroduodenoscopy, infants* = 0 — 3 months old. Note for **b**: *SEEM* Study of Environmental Enteropathy and Malnutrition, *CCHMC* Cincinnati Children’s Hospital Medical Center, *IBD* Inflammatory Bowel Disease, *CBC* complete blood count, *CRP* C-Reactive Protein, *EE* Environmental Enteropathy, * = with a preference to enroll children under 5 years of age

One such previous attempt utilizing duodenal biopsies compared malnourished children in Gambia with healthy UK age-matched children to better understand the pathogenesis underlying this disorder [10]. This study was, however, restricted to morphometric and targeted immunohistochemical analyses for immune cell markers, and did not investigate gene expression associated with the absorptive epithelial layer more broadly. The authors concluded that cell-mediated Th1 response might impair mechanisms of oral tolerance and drive progressive growth failure despite intensive nutritional intervention [10]. Therefore, we propose to supplement routine clinical histologic morphometric analyses with (i) targeted staining to characterize immune cells and the epithelial layer (working with the UVa Biorepository and Tissue Research Facility (BTRF) we have recently established a protocol for triple color immunohistochemical staining), (ii) RNA-Seq analyses to capture a more inclusive EE gut gene expression signature, (iii) gut biopsy DNA-based 16S rDNA characterization of the biopsy-adherent bacterial communities, and (iv) perform microbe:gene association studies (Fig. 3). We hope that these data will provide new insights into both disease pathogenesis and treatment, as well as gut-derived circulating biomarkers for disease severity, which may be assayed in future studies using the banked sera from the larger 400 patient SEEM cohort.

Recently, RNA-Seq and 16S rDNA characterization has been employed to characterize the global pattern of ileal gene expression and the ileal microbial community in treatment-naïve pediatric patients with Crohn’s disease, disease controls with ulcerative colitis, and healthy control individuals. [27] This was followed by a previously established multivariate approach (MaAsLin) [49, 50] to test for associations between selected genes and the microbial taxa. The resultant analyses showed a significant association between expression of components of the APOA1 module and specific *Firmicutes* and *Bacteriodetes* [27].

Furthermore, a multivariate analysis which included disease severity and treatment exposures, showed that Crohn’s patients with the greatest reduction in APOA1 expression at diagnosis were the least likely to achieve clinical remission with current therapies. This suppression of the anti-oxidant lipoproteins in Crohn’s could hence serve as a potential target for future therapies [27].

Similar to the above findings in Crohn’s patients, gene expression studies of duodenal biopsies comparing age-matched controls and celiac patients likewise demonstrate reduction of APOA1 coupled with induction of IFN_γ_ [28]. Together with results described by Campbell et al. [10] showing induction of IFN_γ_ expressing T cells in children with EE in Gambia, these data suggest that this IFN_γ_/APOA1 gene co-expression signature may represent a common pathway of chronic small bowel inflammation and malnutrition.

We plan to test this novel concept by including analysis of duodenal gene expression from disease controls with celiac disease and Crohn’s, together with healthy age-matched controls enrolled at CCHMC. This will provide critical insight into shared and unique features of host epithelial and immune pathogenesis, relative to what are likely to be distinct microbial shifts, across these three disorders in undernourished Pakistani and US children. We anticipate that the induction of IFN_γ_ in EE will in turn be associated with a reduction in apolipoproteins and enterocyte lipid metabolism pathways similar to Crohn’s [27], and celiac disease [28]. Different types of APOA1 interventions are in pre-clinical development for atherosclerosis and inflammatory bowel disease. If we identify a reduction in APOA1 and associated enterocyte lipid metabolic pathways in EE, such treatment may benefit children with EE as well.

### Data analysis plan

This study will result in a vast dataset containing sociodemographic and anthropometric information, as well as biomarkers found in urine, stool, and blood, and endoscopic biopsy results highlighting histopathologic features of both diseased and healthy gastrointestinal tracts.

The WHO Child Growth Standards (WHO Anthro, Geneva, Switzerland) [51] will be used to calculate z-scores, and assess growth both as continuous measures of height-for-age z-score (HAZ), weight-for-age z-score (WAZ) and weight-for-height z-score (WHZ); and as categorized variables of stunting as HAZ < − 2 SD (standard deviation), underweight as WAZ < − 2 SD and wasting as WHZ < − 2 SD. Participant descriptive statistics will be presented as means (standard error, SE) and as frequencies (percentages) for continuous and categorical outcomes, respectively. We will also perform simple linear regression for a specific time point and mixed-effects modeling analysis for repeated measurements to study change in growth trends over the follow-up period.

Mass spectrometry will be used to determine serum and urine bile acid profiles and serum non-essential fatty acid levels, and amino acid profiles. In addition, the cellular fraction of the obtained blood will be utilized for DNA extraction and high-throughput genotyping using Infinium HumanOmniExpressExome [52]. Genotyping will also be used to determine HLA typing of the individual and their associated genetic ethnicity. Given the similarities between EE and celiac disease, it will be of interest to determine whether there is also a common HLA genotype associated with EE [53]. Both environmental and genetic factors [54], play roles in gut microbial composition, therefore, we will control for genetic variants (i.e. HLA [55], FUT2 [56]) in some of the planned microbial and gene expression analyses.

We will use a variety of computational/statistical approaches to assess the extent to which expression of various genes and their associated metabolic/signaling pathways in duodenal biopsies in children with celiac disease, Crohn’s disease, and EE overlap or differ. We will approach this using several statistical methods; PCA plots; Venn diagrams of the differently expressed genes between celiac disease vs. controls, EE vs. controls, and Crohn’s disease vs. controls; and associated pathway analyses. A significant overlap in pathways could provide insights into pathogenesis and new treatment approaches, such as tight junction modulation, which are in development for celiac disease [57]. Gene signatures distinct for EE would further provide a promising source of future therapeutic targets and initial tissue-level validation of promising biomarkers that would be useful for predicting growth and powering studies to test future interventions against EE. We also plan to compare proximal small intestinal duodenal biopsies with distal ileal biopsies in the Crohn’s cohort to determine how well the duodenal pattern of gene expression reflects the ileum.

Analyses of biopsies from both Crohn’s disease and celiac disease patients will also provide us the opportunity to test for the relationship between the microbial community and gene expression signatures. We will test for associations between taxa of the duodenal microbial community and specific clinical and gene expression metadata using Multivariate Analysis by Linear Models (MaAsLin) as has been described in prior studies [27, 49, 50]. A comprehensive description of this analysis method has been published online [58]. In short, for each arcsine square root transformed microbial feature, a model is selected from metadata using gradient boosting (gbm package [59]). Covariates in the selected model are then evaluated controlling for potential confounders using a general linear model. Additionally, confounders will also be assessed via construction of a causal pathway. Multiple comparisons over factor levels will be adjusted using a Bonferroni correction, and multiple hypothesis tests over all clades and metadata will be adjusted with a false discovery rate [60].

All significant (α < 0.05) associations will be investigated in the analysis: clinical phenotype (including severity of wasting), stool and duodenal enteropathogen burden, endoscopic inflammation, histologic severity, and selected duodenal differentially expressed genes such as IFN_γ_ and APOA1. We plan to control for age, gender, and body mass index (as a measure of nutritional status) in our analysis. These analyses will identify specific duodenal microbial taxa associated with differences in IFN_γ_ and/or APOA1 gene expression across the three disorders tested; EE, Crohn’s disease, and celiac disease.

Next, analyses of mRNAseq performed on biopsy tissues will be carried out using polyA-RNA selection, fragmentation, cDNA synthesis, adaptor ligation, and library preparation with TRUSeq RNA Sample preparation (Illumina, San Diego, CA, USA). Paired-end 75 bp sequencing will be performed using the Illumina HiSeq 2000 in the CCHMC NIH-supported Digestive Health Center with a minimum depth of 20 million reads per sample. Reads will be aligned using TopHat [61]. The aligned reads will be quantified by Avadis NGS software (Version 1.3.0, Build 163,982 Strand Scientific Intelligence, Inc., San Francisco, CA, USA) using Hg19 as the reference genome and RPKM as an output. The DESeq algorithm will be used for RPKM normalization within Avadis NGS software. Two more recently applied alternative approaches for quantification will also be assessed using kallisto [62] and eXpress [63].

For RNA-Seq expression and gene enrichment analysis samples will be stratified into specific clinical subgroups including control, EE, celiac disease, and Crohn’s disease. For some analyses the EE group will be further sub-divided into those with an identified infection (e.g. *Giardia, H. pylori*) and those without an identified infectious etiology. In addition, we plan to stratify the Crohn’s patient samples by their location to assess expression differences between proximal (duodenal) and distal (ileum) small bowel expression.

Other potential sub-stratification of the EE group will be based on CRP level, histologic severity, response to rotavirus infection (by measuring rotavirus IgA levels), and weight/height outcomes. Differentially expressed genes of the above stratified groups will be determined by the Audic Claverie method using the Benjamini-Hochberg false discovery rate correction (FDR 0.05) and analyzed for fold change differences. Normalized intensity values will be used for patterns of gene expression. Pearson correlation based on trend and rate of change will be performed for IFN_γ_ and APOA1 gene expression across defined groups for correlation coefficient of 0.98 < |r| < 1.

ToppGene [64], ToppCluster [65], and IPA (Ingenuity Systems) software will be used to test for functional annotation enrichment analyses of upstream regulators, immune cell types, pathways, phenotype, and biologic functions. Functional annotation enrichment analyses for immune cell type enrichments will be characterized using the Immunological Genome Project data series through ToppGene. Visualization of the functional networks will be obtained using Cytoscape v.3.02 [66].

Lastly, an anticipated challenge in interpreting clinical biopsies from EE and celiac disease will be the possible histopathological overlap between these distinct but related enteropathies [10, 22]. In light of this, we propose to use duodenal biopsy data (converted to whole slide images using Leica SCN400 slide scanner [Meyer Instruments, Houston, TX] and Olympus VS120 Virtual Slide Microscope [Olympus Corporation Inc., Center Valley, Pennsylvania]) from EE, celiac disease and normal healthy biopsy from patients recruited in SEEM and archival duodenal biopsies from the UVa BTRF as data input for a deep learning image analysis algorithm. Deep learning, or machine learning, has been said to be the natural extension to our current statistical analysis [67] especially in the context of multiomic data as in SEEM, and is at the forefront of advances in both technology and medicine [68]. We will use a subtype of deep learning known as Convolutional Neural Networks (CNNs) [69], to detect morphological distinguishing histological features between disease phenotypes. We will also couple our CNN layers with a deconvolution layer [70, 71], and a Gradient Class Activation Map layer [72], which will allow us to trace back high activation features to the corresponding biopsy. This will allow us to enhance the detection of pathologic morphological features that can help distinguish between celiac disease and EE, both when compared to each other and also when compared to healthy duodenal tissue. Furthermore, in addition to applying the EEDBI scoring system, we will use this deep learning driven approach to correlate high activation features to identify multiomic patterns not identified by traditional pathology scoring.

**Fig. 4:**
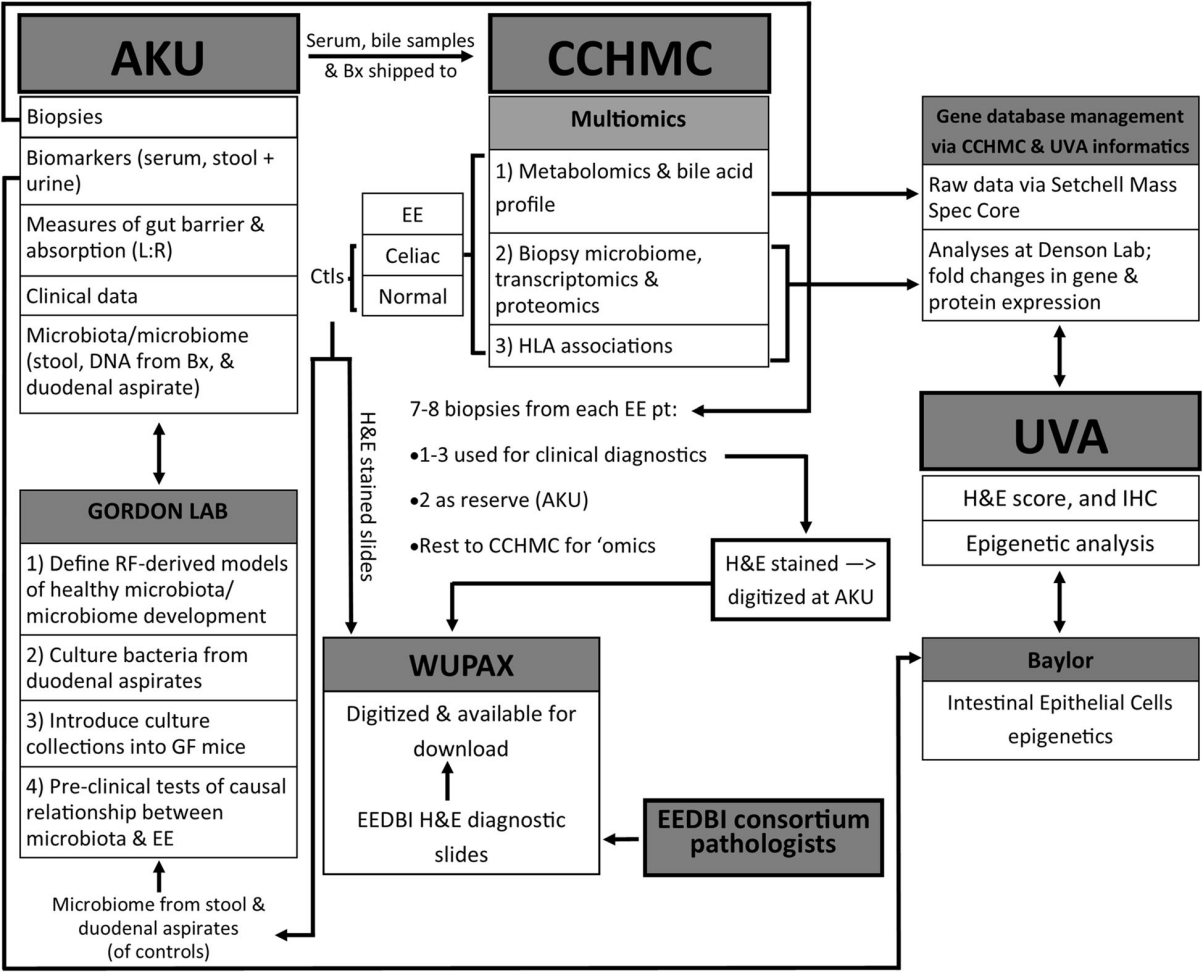
Framework of data flow in SEEM. Description of how data will be transferred between institutions and a summary of the samples/analyses conducted at each institute. Note: *AKU* Aga Khan University, *L:R* Lactulose:Rhamnose ratio, *Bx* biopsy, *EE* Environmental enteropathy, *CCHMC* Cincinnati Children’s Hospital Medical Center, *HLA* Human Leukocyte Antigen, *UVA* University of Virginia, *‘omics* multiomics, *H&E * Haemotoxylin and Eosin, *IHC* Immunohistochemistry, *EEDBI* Environmental Enteric Dysfunction Biopsy Initiative, *WUPAX* Washington University Digital Pathology Exchange, *GF* Germ Free

The overall schedule for enrolment, interventions and assessments, including UGI endoscopy and biopsies, is described in Fig. 3; and the data transfer between each collaborating institution is outlined in Fig. 4. To ensure optimal outcomes from this ambitious undertaking, we designed SEEM with monitoring and quality control measures, timelines for milestones, anticipation of challenges, and consideration of ethics and data dissemination.

### Safety measures and preparation for adverse events

One of our major goals was to optimize safety for UGI endoscopies, especially given our LMIC study setting. Although never without elements of risk during the procedure and anesthesia, endoscopy is a very safe procedure when conducted by trained experienced personnel in a well-equipped facility. We have safety data from our own pilot EE phase 1 study in which we successfully performed endoscopy on 11 children with a median (Q1 – Q3) age of 22 (20–23) months (14). There is also data from studies conducted in Gambia [10] where children underwent endoscopy under anesthesia, and Zambia [73] where malnourished children underwent endoscopies with collection of duodenal biopsy samples under anesthesia. Neither of these studies reported any endoscopy-related adverse events during or after the procedures.

Additionally, several questions arose in the development and implementation of our Phase 1 EE study and subsequently in SEEM, all of which were reviewed by the Ethical Review Committee (ERC) at AKUH for discussion and resolution. For example, adverse events that may arise during endoscopy were a major concern; to mitigate issues, investigators have developed a priori definitions, assessment criteria and action guidelines including an overnight stay in Karachi prior to return to the subject’s village Matiari. Endoscopy of eligible children will be performed at the AKUH (accredited by the Joint Commission International, IL, USA since July 2006 [74, 75]) where the highest safety standards at par with hospitals in high-income country settings are met including access to a pediatric intensive care unit, pediatric surgeons and dedicated clinical dieticians. Of note, children who fail to respond to nutritional rehabilitation are evaluated by a team of physicians including a trained pediatric gastroenterologist (KS). A qualified anesthetist attends every endoscopy procedure to assess and administer steps as required for sedation. The participant’s oxygen saturation level, pulse rate and blood pressure are continuously monitored throughout the procedure. Resuscitation measures and complete pediatric Intensive Care Unit (ICU) support remain available during the procedure for immediate resuscitation if necessary. Clinical findings from the biopsies (e.g. presence of gastritis, H pylori infection, Giardiasis or diagnosis of celiac disease) are made available as soon as possible so that appropriate treatment can be undertaken. Additionally, morbidity and poor appetite also represent a big challenge; by providing close follow-up, proper counseling and by practicing supportive feeding techniques, this issue can be resolved. Our field team in Matiari is led by two physicians registered by the Pakistan Medical and Dental Council (PMDC), who will oversee these challenges, along with access to a panel of North American trained pediatric subspecialists (SAA - pediatric infectious disease; KS, SS, and SRM - pediatric gastroenterology) for additional expertise as needed.

Other expected adverse events for this protocol are those related to the endoscopy/biopsy procedure that do not qualify as a serious adverse event (SAE) and those associated with phlebotomy and ingestion of lactulose/rhamnose solution. Both serious and non-SAEs are assessed for their severity, their relationship to study participation and the actions taken and their outcomes. All SAEs are being reported to the AKUH ERC within 24 h of the site’s awareness of the event. In the event that medical care is required outside of the protocol, all necessary and available treatments are provided, free of cost.

### Monitoring and quality control

With regards to monitoring and quality control, we took several measures to ensure best practices for identifying mislabeling of data. Sample mislabeling has been known to occur, for example mislabeling gender. Such mislabeling was identified when we found that the inferred gender based on genotype did not correlate with actual gender reported on clinical metadata. In light of this samples were rechecked and re-labeled correctly by our study team. A quality control algorithm was developed that combines base calling from the biopsy mRNAseq data and tests its genetic concordance with genotyping of the DNA extracted from the blood [76]. In order to automate this mislabeling, it will be important to apply a similar quality control approach in our study, where incorrect linkage of duodenal expression data to clinical data could result in significant variation in the results. This work will be supported by the same infrastructure at CCHMC currently being used for multi-center inflammatory bowel disease cohort studies. This includes Gene and Protein Expression and Bioinformatics cores of the National Institute of Health (NIH) – supported by CCHMC Digestive Health Center.

### Ethical clearance

The SEEM study protocol has been approved by the AKUH ERC (Protocol 3836-Ped-ERC-15), which is an independent regulatory authority equivalent to Institutional Review Board (IRB). SEEM has also previously received ethical approval from the Cincinnati Children’s Hospital Medical Center (CCHMC, Study ID 2016–0387), and met ethical standards during an audit conducted by the Aga Khan University for an ethical compliance review (Study ID 2446). Collection of specimens for studies on the gut microbiome was approved by the Washington University Human Research Protection Office (IRB ID 201111065). The University of Virginia Institutional Review Board has also approved SEEM (UVa-IRB, Study ID 19856) for the purpose of intestinal tissue triple color immunohistochemistry via the UVa BTRF.

All medical and research ethics will be followed during the interaction with each participant enrolled in SEEM, and also for any and all data collected from them. After complete disclosure, a signed informed consent (Additional file 2) will be obtained from each participant’s parent or legal guardian. The consent will be obtained, preferably, where the participant resides. If the parent(s)/guardian agree to participate in the study, the consent form must be signed or an impression of their thumb must be provided. The investigator and a witness will also sign the form. For endoscopy, a separate consent form is used and the same procedure is followed. The consent form for endoscopy will clearly and fully describe all aspects of the process, including the risks related with the procedure. No information is remained withheld from the participant.

## Discussion

Given the operational and ethical limitations for safely obtaining intestinal biopsies from children in resource-poor settings, there have been few detailed investigations of human tissue in this vulnerable group for whom reversal of EE would be extremely beneficial [14, 17–21]. Furthermore, EE biomarkers studied in different settings have not been correlated with the gold standard of histopathology [17, 18, 21]. SEEM is designed to better understand the pathophysiology, predictors, biomarkers, and potential management strategies of EE to inform strategies to eradicate this debilitating pathology. SEEM will help define EE, however this definition will potentially be biased by the presumption of EE in children in whom we do not identify an acute or chronic gut infection or other GI pathology. Hence, it will be important to compare our results with those of other biopsy-based EE studies currently underway (which have used different enrolment criteria, but equivalent histopathological assessment and scoring) and to provide an improved or modified definition of EE that captures the full spectrum of the disease.

The data, results and other findings resulting from this study will be published only after approval by a committee consisting of the investigators of the protocol. The International Committee of Medical Journal Editors guidelines will be used to establish authorship on papers [77]. As of September 2018, participant enrollment has been completed.

## Additional files

Additional file 1: **Figure S1.** Urine and fecal sample collection protocol. Panel A describes the urine collection protocol followed by the community health workers (CHWs), and Panel B describes the fecal collection protocol followed by the CHWs for instant transport of fecal samples in a dry shipper for long term storage and preservation for microbiome analysis. Please note: *L:R* Lactose Rhamnose ratio, *mL* milliliter, *CHW* Community Health Worker, *AKU* Aga Khan University, *IDRL* Infectious Diseases Research Laboratory.

Additional file 2: Informed consent forms for recruitment of healthy and malnourished children.

## Data Availability

Not applicable.

## Abbreviations

AKUH: Aga Khan University Hospital
BTRF: Biorepository and Tissue Research Facility
CCHMC: Cincinnati Children’s Hospital Medical Center
CHWs: Community health workers
CNNs: Convolutional Neural Networks
EE: Environmental Enteropathy
EEDBI: Environmental Enteric Dysfunction Biopsy Initiative
EPI: Expanded program on immunization
ERC: Ethical Review Committee
FITC: Fluorescein Isothiocyanate
H&E: Hematoxylin and eosin
HAZ: Height for age Z score
ICU: Intensive care unit
IDRL: Infectious Diseases Research Laboratory
IEC: Intestinal epithelial cells
IRB: Institutional Review Board
L:R: Lactose:rhamnose
LMIC: Low- and middle income country
MaAsLin: Multivariate Analysis by Linear Models
NIH: National Institute of Health
PMDC: Pakistan Medical and Dental Council
RPKM: Reads per kilobase per million mapped reads
SAE: Serious adverse event
SAM: Severe acute malnutrition
SEEM: Study of Environmental Enteropathy and Malnutrition
UGI: Upper gastrointestinal
UVa: University of Virginia
WHO: World Health Organization
WHZ: Weight for height Z score
WUSTL: Washington University in St. Louis
