# Supplementary figures and images for: Study of Environmental Enteropathy and Malnutrition (SEEM) in Pakistan: protocols for biopsy based biomarker discovery and validation
Source: BMC Pediatr. 2019 Jul 22;19:247. doi: 10.1186/s12887-019-1564-x (PMC6643315; doi:10.1186/s12887-019-1564-x)

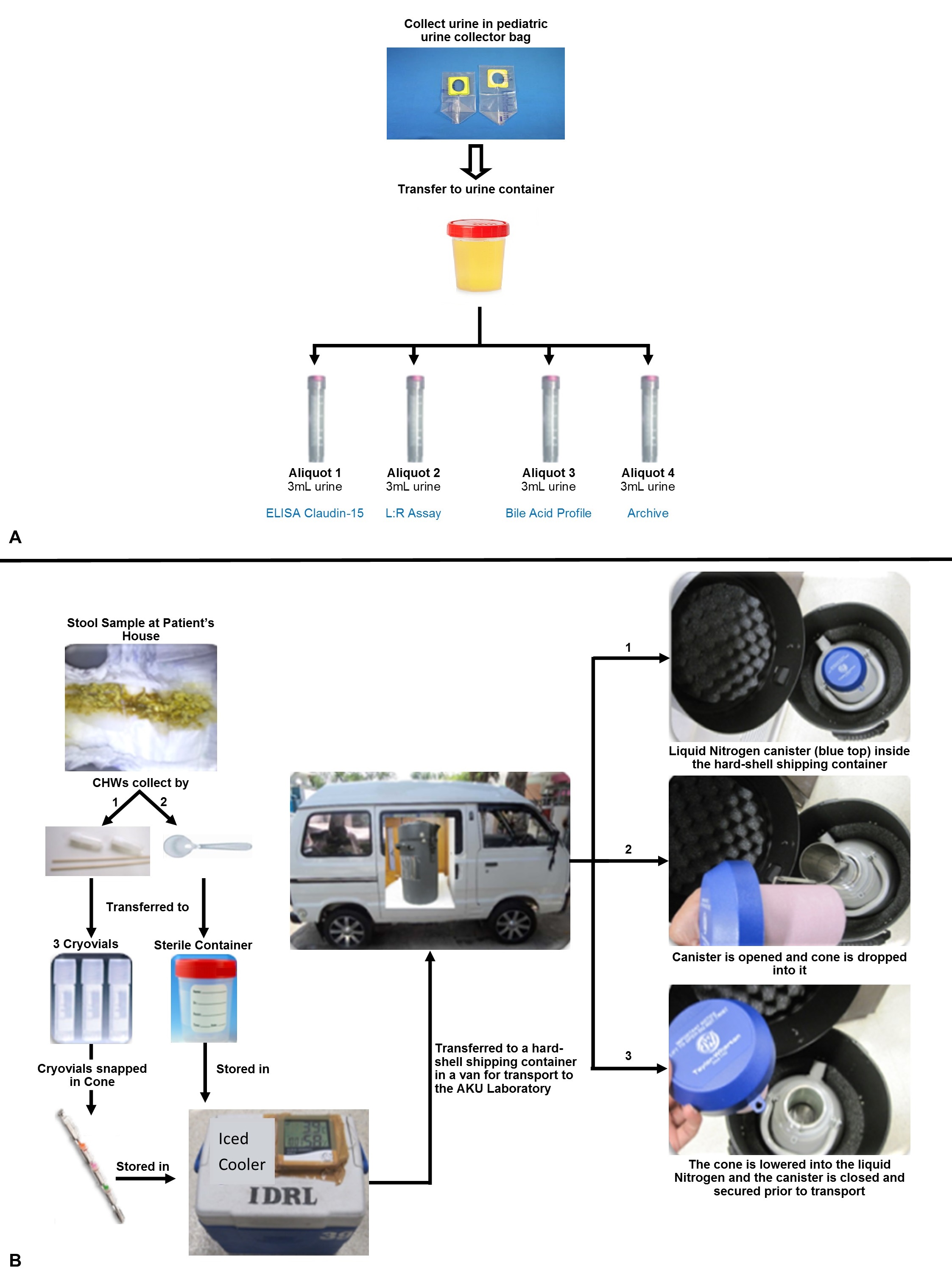

Supplement: Supplementary file 1 — Figure S1. Urine and fecal sample collection protocol. Panel A describes the urine collection protocol followed by the community health workers (CHWs), and Panel B describes the fecal collection protocol followed by the CHWs for instant transport of fecal samples in a dry shipper for long term storage and preservation for microbiome analysis. Please note: L:R Lactose Rhamnose ratio, mL milliliter, CHW Community Health Worker, AKU Aga Khan University, IDRL Infectious Diseases Research Laboratory. [file 12887_2019_1564_MOESM1_ESM.jpg]
