# Supplementary material for: Study of Environmental Enteropathy and Malnutrition (SEEM) in Pakistan: protocols for biopsy based biomarker discovery and validation
Source: BMC Pediatr. 2019 Jul 22;19:247. doi: 10.1186/s12887-019-1564-x (PMC6643315; doi:10.1186/s12887-019-1564-x)
Supplement: Supplementary file 2 — Informed consent forms for recruitment of healthy and malnourished children. [file 12887_2019_1564_MOESM2_ESM.docx]

**PARENTAL PERMISSION DOCUMENT**

**Informed Consent form from Healthy control child**

**Child ID:**

| Project Title: Study of Environmental Enteropathy and Malnutrition in Pakistan (SEEM-Pakistan) | Project ID: OPP1138727 |
| --- | --- |
| Sponsor: BMGF | ERC Ref #: 3836-Ped-ERC-15 |
| Principal Investigator: Dr. Asad Ali | Organization: Aga Khan University, Department of Paediatrics & Child Health |
| Project Location: Matiari | Phone: 34864955-021 |

**Introduction**

We at the department of Paediatrics and Child health at Aga Khan University are conducting a research study to develop new techniques to diagnose and predict malnutrition in children. This project, called the SEEM study (Study of Environmental Enteropathy and Malnutrition in Pakistan), is a partnership involving Cincinnati Children’s Hospital and Washington University School of medicine. This project is funded by Bill and Malinda Gates Foundation. Project ID is OPP1138727.

**Why is the study being done?**

The consequences of malnutrition if no action is taken are enormous. The greatest functional consequences of malnutrition for children are increased risk of illness, and death; and for those who survive mental impairment and reduced capacity to produce and contribute to the economy of the country. These consequences of malnutrition are often not fully appreciated because they are hidden Large number of children in our community are malnourished. Effective interventions to prevent and treat malnutrition in children will reduce childhood mortality and lead to improved long-term productivity of the population

**Why is your baby being asked to participate?**

We are going to enrol your child in SEEM study, in which our research appointed health worker is visiting your household to collect general information about your child. She is also doing anthropometry to assess whether your child is malnourished or healthy.

Some healthy babies who are being regularly visited are selected at random (i.e., by chance) to have same tests that we are conducting on malnourished children.

**What are you being asked to do?**

At 3-6 months, your child will be assessed clinically for malnutrition. Since your child is healthy and got selected, we will collect your child’s blood (approximately 3 ml) and urine samples at 3-6 and 9 months. Breast milk sample from mother will be collected at 3-6 month. At nine months of your child’s age, a special urine test will be conducted in which your child will be offered half a cup of sugar solution and then his/her urine will be collected afterwards. Stool samples will be collected on monthly basis.

All of the samples will be first sent to the Matiari field laboratory for testing and then to Aga Khan Laboratory at Karachi. However, some of your baby’s specimen may be send to the Laboratories in the USA. Results of these tests will be reported to you if they are directly related to decisions you or a physician would make about your baby’s health Your child will be followed by our workers for up to 2 years after enrolment. If she finds any illness in your child during her follow up visits or your child becomes sick then our mobile medical team will assess your child and will give medical treatment as required.

**What are the anticipated benefits?**

There are no direct benefits to your baby by joining this part of the study. The main benefit of the study is for your community, by helping to find the causes and risk factors of Malnutrition in Pakistan**.** This information will be used to design better ways to prevent and treat Malnutrition.

**What are the possible risks or discomforts?**

There are no major risks to your baby by joining this component of the study. Collection of the samples of blood may cause minor discomfort, bleeding or bruising. There is a very small risk of infection. We will minimize such problems by cleaning the skin and using disposable syringes and needles. It is safe for your baby to give the small amounts of blood that we will collect. Collection of the urine and stool samples may cause minor discomfort, but they are very safe and will be collected by our experienced staff.

#### Right of refusal to participate and withdrawal

You have the right to decide for your participation into the study. You may refuse participation without any loss of benefit for which you are otherwise entitled to. Your enrolled child will receive the same standard care and treatment which is considered best, irrespective of your decision to participate in the study. You may also withdraw any time from the study without any adverse effect on management of the concerned disease. You are also at will to answer all, some or none of the questions if you do not feel comfortable with them.

**Protecting data confidentiality**

Your baby’s personal information will remain confidential. Only study staff will have access to study forms and will be destroyed 10 years after the end of this study. We will not use or publish your name when we discuss the results of this study in public

The PI will be responsible for ensuring confidentiality of all material collected.

**Blood, urine and stool, samples from your baby and mother’s milk may be stored to use in future research.**

The specimens may be used to learn about the body and to improve health. They may also be used to develop new tests related to malnutrition which are beyond the scope of this study at present. If we complete our research or cannot store the specimens any longer, we will destroy them.

You can choose to refuse the storage of your baby’s samples. In that case we will destroy the left over specimen after completing the SEEMS specific tests.

- My baby’s blood/urine/stool sample(s) **and mother’s milk** may be used for future research on child’s health. _____ Yes _____ No

#### Available source of information

Any further questions you have about this study will be answered by the Principal Investigator **Dr. Syed Asad Ali** at Department of Paediatrics, Aga Kahn University, Stadium Road, P.O. Box 3500, Karachi-74800, and Pakistan on phone # 021-34864955.

**What does your signature (or thumbprint/mark) on this consent form mean?**

Your signature (or thumbprint/mark) on this form means:

- You have been informed about this study’s purpose, procedures, possible benefits and risks.
- You have been given the chance to ask questions before you sign.
- You have voluntarily agreed that your baby can be in this study.
- You have agreed to report immediately to the research staff if your baby has any unexpected or unusual symptoms.

**Name of child and their Legally Authorized representative name & signature**

Name of child _________________________

**Legally authorized representative** name _______________

Signature ______________Date_____________

**Name & signature of Person Obtaining Consent**

Name ________________________ Signature ___________________ Date______________

**Name & signature of Witness (If Legally Authorized representative is unable to Read or Write)**

Name ________________________ Signature ___________________ Date______________

**PARENTAL PERMISSION DOCUMENT**

**Informed Consent form for recruitment of malnourished child**

**Child ID:**

| Project Title: Study of Environmental Enteropathy and Malnutrition in Pakistan (SEEM-Pakistan) | Project ID: OPP1138727 |
| --- | --- |
| Sponsor: BMGF |  |
| Principal Investigator: Dr. Asad Ali | Organization: Department of Paediatrics & Child Health, Aga Khan University, , Karachi, Pakistan |
| Project Location: Matiari | Phone: 34864955-021 |

**Introduction**

**Study Title:**

We at the department of Paediatrics and Child health at Aga Khan University are conducting a research study to develop new techniques to diagnose and predict malnutrition in children. This project, called the SEEM study (Study of Environmental Enteropathy and Malnutrition in Pakistan), is a partnership involving Cincinnati Children’s Hospital and Washington University School of medicine. This project is funded by Bill and Malinda Gates Foundation. Project ID is OPP1138727.

**Why is the study being done?**

The consequences of malnutrition if no action is taken are enormous. The greatest functional consequences of malnutrition for children are increased risk of illness, and death; and for those who survive mental impairment and reduced capacity to produce and contribute to the economy of the country. These consequences of malnutrition are often not fully appreciated because they are hidden. Large number of children in our community are malnourished. Effective interventions to prevent and treat malnutrition in children will reduce childhood mortality and lead to improved long-term productivity of the population

**Why is your baby being asked to participate?**

We are going to enrol your child in SEEM study, in which our research appointed health worker is visiting your household to collect general information about your child. She also is doing anthropometry to assess whether your child is malnourished or healthy. Since your child is malnourished, we will enrol your child in our study.

**What are you being asked to do?**

At six months of age, we will first do the educational sessions in order to improve your child nutritional status. If your child is still malnourished at 9 months of age, we will provide nutritional supplements for 4 weeks. If after 4 weeks of nutritional intervention your child gets better, we will continue the nutritional intervention for further few weeks.

However, if after 4 weeks your child fails to improve, then a team of paediatricians at Aga khan University hospital will assess your child and will decide on clinical grounds whether your child needs to undergo additional work up to identify possible reasons of malnutrition, which may include upper gastrointestinal endoscopy and biopsy. Separate permission will be taken for this procedure.

We are requesting you to participate in this study, where we will collect your child’s blood (approximately 3 ml), urine & stool samples at 3-6 and 9 months to identify reasons of malnutrition in children. All of the samples will be first sent to the Matiari field laboratory for testing and then to Aga Khan Laboratory in Karachi. Some of your our baby’s specimen may be send to the Laboratories in the USA. Results of these tests will be reported to you if they are directly related to decisions you or a physician would make about your baby’s health. Your child will be followed by our workers for up to 2 years after enrolment. If she finds any illness in your child during her follow up visits or your child becomes sick then our mobile medical team will assess your child and will give medical treatment as required.

**What are the anticipated benefits?**

Free treatment and evaluation will be done for your child. Closed observation, work up for malnutrition and nutritional rehabilitation will also be provided as well. If still no cause of malnutrition is found, treatment according latest guidelines will be provided.

**What are the possible risks or discomforts?**

There are no major risks to your baby by joining this component of the study. Collection of the samples of blood may cause minor discomfort, bleeding or bruising. There is a very small risk of infection. We will minimize such problems by cleaning the skin and using disposable syringes and needles. It is safe for your baby to give the small amounts of blood that we will collect. Collection of the urine and stool samples may cause minor discomfort, but they are very safe and will be collected by our experienced staff

#### Right of refusal to participate and withdrawal

You have the right to decide for your participation into the study. You may refuse participation without any loss of benefit for which you are otherwise entitled to. Your enrolled child will receive the same standard care and treatment which is considered best, irrespective of your decision to participate in the study. You may also withdraw any time from the study without any adverse effect on management of the concerned disease. You are also at will to answer all, some or none of the questions if you do not feel comfortable with them

#### Confidentiality

**Protecting data confidentiality:**

Your baby’s personal information will remain confidential. Only study staff will have access to study forms and will be destroyed ten years after the end of this study. We will not use or publish your name when we discuss the results of this study in public. The PI will be responsible for ensuring confidentiality of all material collected.

**Blood, urine and stool, samples from your baby may be stored to use in future research.** The specimens may be used to learn about the body and to improve health. They may also be used to develop new tests related to malnutrition which are beyond the scope of this study at present. If we complete our research or cannot store the specimens any longer, we will destroy them.

You can choose to refuse the storage of your baby’s samples. In that case we will destroy the left over specimen after completing the SEEMS specific tests.

- My baby’s blood/urine/stool sample(s) may be used for future research on child’s health. _____ Yes _____ No

#### Available source of information

Any further questions you have about this study will be answered by the Principal Investigator **Dr Syed Asad Ali** at Department of Paediatrics, Aga Kahn University, Stadium Road, P.O. Box 3500, Karachi-74800, and Pakistan on phone # 34864955.

**What does your signature (or thumbprint/mark) on this consent form mean?**

Your signature (or thumbprint/mark) on this form means:

- You have been informed about this study’s purpose, procedures, possible benefits and risks.
- You have been given the chance to ask questions before you sign.
- You have voluntarily agreed that your baby can be in this study.
- You have agreed to report immediately to the research staff if your baby has any unexpected or unusual symptoms.

**Name of child and their Legally Authorized representative name & signature**

Name of child _________________________

**Legally authorized representative** name _______________

Signature ______________Date_____________

**Name & signature of Person Obtaining Consent**

Name ________________________ Signature ___________________ Date______________

**Name & signature of Witness (If Legally Authorized representative is unable to Read or Write)**

Name ________________________ Signature ___________________ Date______________

**PARENTAL PERMISSION DOCUMENT-addendum**

**Informed Consent form for recruitment of malnourished child**

**Child ID:**

| Project Title: Study of Environmental Enteropathy and Malnutrition in Pakistan (SEEM-Pakistan) | Project ID: OPP1138727 |
| --- | --- |
| Sponsor: BMGF |  |
| Principal Investigator: Dr. Asad Ali | Organization: Department of Paediatrics & Child Health, Aga Khan University, Karachi, Pakistan |
| Project Location: Matiari | Phone: 021-34864955 |

**What are you being asked to do?**

As your child is already enrolled in the study, we would like to take two additional tests including breastmilk sample from mother and LR urine test from your child. Breastmilk test is safe and is being done to know the cause of malnutrition in your child. However, during this process of collecting breast milk may cause minor discomfort to mother. At nine months of your child’s age, a special urine test will be conducted in which your child will be offered half a cup of sugar solution and then his/her urine will be collected afterwards. Sugar is safe and has no side effects and but this test can cause minor discomfort to your child. If you allow us to perform these two additional tests, only then we will collect these samples.

**Name of child and their Legally Authorized representative name & signature**

Name of child _________________________

**Legally authorized representative** name _______________

Signature ______________Date_____________

**Name & signature of Person Obtaining Consent**

Name ________________________ Signature ___________________ Date______________

**Name & signature of Witness (If Legally Authorized representative is unable to Read or Write)**

Name ________________________ Signature ___________________ Date______________
